# Clinical characteristics and prognostic analysis of Lung Cancer patients with Hypercoagulability: A single-center, retrospective, real-world study

**DOI:** 10.7150/jca.46600

**Published:** 2021-03-19

**Authors:** Yunfei Ma, Guangda Li, Xiaoxiao Li, Yu Gao, Tongjing Ding, Guowang Yang, Yi Zhang, Jiayun Nian, Mingwei Yu, Xiaomin Wang

**Affiliations:** 1 Beijing Hospital of Traditional Chinese Medicine, Capital Medical University, Beijing, 100010, China.; 2 Beijing University of Chinese Medicine, Beijing, 100029, China.

**Keywords:** lung neoplasms, hypercoagulability, clinical characteristics, prognosis, real-world study

## Abstract

**Objective:** We explored the clinical regularity and prognosis of lung carcinoma (LC) patients with hypercoagulability, which is often associated with the occurrence and development of tumors.

**Methods:** This retrospective study analyzed 624 LC patients diagnosed from 2010-2017 in the Beijing Hospital of Traditional Chinese Medicine, Capital Medical University, China. Kaplan-Meier analysis was used to estimate survival and the log-rank test was used to identify differences in survival between groups. The predictive power of a hypercoagulation model was tested using receiver operating characteristic (ROC) curve analysis. Univariate and multivariate Cox regression analyses were performed to explore independent factors associated with survival**.** A logistic regression model was used to explore factors related to hypercoagulability. The diagnostic power of relevant influencing factors on hypercoagulability was tested using ROC curve analysis.

**Results:** Of 624 patients in the study, 161(25.8%) had hypercoagulability and 463 did not (normal group). The overall survival (OS) of the hypercoagulability group was significantly lower than the normal group (*P* < 0.0001). The ROC curve showed that the predictive power of the hypercoagulability model was better than that of a single coagulation indicator (*P* < 0.01). Both univariate and multivariate Cox regression analyses showed that hypercoagulability was an independent factor affecting the prognosis of LC (*P*<0.0001). The results of the logistic regression analysis showed that clinical stage (*P* < 0.05), cytokeratin 19 fragment (Cyfra211) (*P* < 0.05), and the platelet-to-lymphocyte ratio (PLR) (*P* < 0.05) were positively correlated with hypercoagulability. When combining clinical stage, Cyfra211, and the PLR to predict hypercoagulability, the area under the ROC curve was 0.797 (*P* < 0.01).

**Conclusions:** In LC, hypercoagulability is an independent factor associated with poor OS and could be a prognostic factor.

## Introduction

Lung carcinoma (LC) is the leading cause of cancer-related death. Of the 9.56 million people who died of cancer in 2018, 1.76 million (18.4%) had LC 1. With the advance of targeted therapy and immunotherapy, great progress has been made in the treatment of LC, but the 5-year survival rate of LC is still dismal 2,3. Therefore, the ability to identify effective prognostic factors for predicting the clinical outcomes of LC is critical to making treatment decisions.

Hypercoagulability is a state of vascular endothelial cell injury, decreased anticoagulation function, and decreased tissue fibrinolytic activity caused by a variety of factors, which lead to increased coagulation of blood. Hypercoagulability in cancer patients is closely associated with the activation of the coagulation system and an increased number of platelets (PLTs). Some studies have demonstrated that hypercoagulability can affect the biological behavior of tumor cells, including promoting proliferation, invasion, and immune escape 4,5. The relationship between coagulation functions and malignancy has been extensively studied 6-8, revealing an association between the prognoses of LC patients and single coagulation-related factors, including levels of PLTs, D-dimer, and fibrinogen (FIB). However, the results of these studies are inconsistent. A multicenter prospective study showed a significant association between an elevated PLT count and poor progression-free survival (PFS), whereas levels of FIB and D-dimer were not significant prognostic factors 9. Hong et al. 10 and Wu et al. 11 suggested that an elevated PLT count was not a prognostic factor of LC. Jiang et al. 12 reported that elevated D-dimer significantly predicted poor survival of LC.

In order to comprehensively evaluate the prognosis of patients with LC, we combined multiple coagulation indicators and constructed a hypercoagulation model based on the study of Yang et al. 13 to evaluate the prognostic value of hypercoagulability and explore the clinical characteristics of LC patients with hypercoagulability. The goal of our study was to provide a new perspective for better understanding of the association between hypercoagulability and prognosis in LC.

## Methods

### Study design

This was a single-center, retrospective, real-world study. The Information Center of the Beijing Hospital of Traditional Chinese Medicine, affiliated to the Capital Medical University (BHTCM) assisted in collecting the information of LC patients in the Hospital Information System (HIS) database. All data were from the first-admission evaluations in our hospital. In the BHTCM HIS system, patients with LC who visited the hospital from January 2010 to December 2017 were screened through the first page of medical records. The search terms included the following: “neoplasms,” “lung,” “lung neoplasm,” “carcinoma,” “non-small-cell lung,” “carcinoma, bronchogenic,” “adenocarcinoma of lung,” “carcinoma, squamous cell,” “small cell lung carcinoma”, “carcinoma, large cell,” and “pulmonary nodule.” Dates of patient death were obtained from the Beijing Centers for Disease Control and Prevention, China.

### Participants

All patients in this study were admitted to the BHTCM from January 2010 to December 2017. The inclusion criteria were a pathological diagnosis of LC, age≥18, and at least two indicators of coagulation function. The exclusion criteria were other tumors, coronary heart disease, cerebral infarction, hemopathy, acute inflammatory reaction, non-tumor-related surgery within the last month, and administration of drugs affecting blood coagulation or anticoagulant function within the last month. Hypercoagulability was defined as meeting at least two of the following criteria: elevated FIB levels, shortened prothrombin time, shortened activated partial thromboplastin time, elevated D-dimer levels, and increased PLTs 13. This study was approved by the institutional review board (2017 BL-087-03).

### Data collection

Clinically relevant data for the first admission of all patients, including general patient information, pathological characteristics, and relevant examinations, were collected by oncology professionals.

### Statistical analysis

Data were exported from EpiData version 3.1 (EpiData Association, Odense, Denmark) to SPSS Version 22.0 (IBM, Armonk, NY, USA) for analysis. Descriptive statistics were performed for patient characteristics, pathological features, and relevant examinations. Quantitative variables were expressed as the mean ± standard deviation, and comparisons were performed by the Mann-Whitney U test. Categorical variables were expressed as N (%), and the chi-square test was used for comparison. Kaplan-Meier analysis was used to estimate survival, and the log-rank test was used to identify differences in survival between groups. The predictive power of a model was tested using receiver operating characteristic (ROC) curve analysis. Univariate and multivariate Cox regression analyses were performed to explore independent factors associated with survival. A logistic regression model was used to explore factors related to hypercoagulability. The diagnostic power of relevant influencing factors on hypercoagulability was tested using ROC curve analysis. A *P*-value < 0.05 was considered statistically significant.

## Results

### Patient characteristics

**Figure 1:**
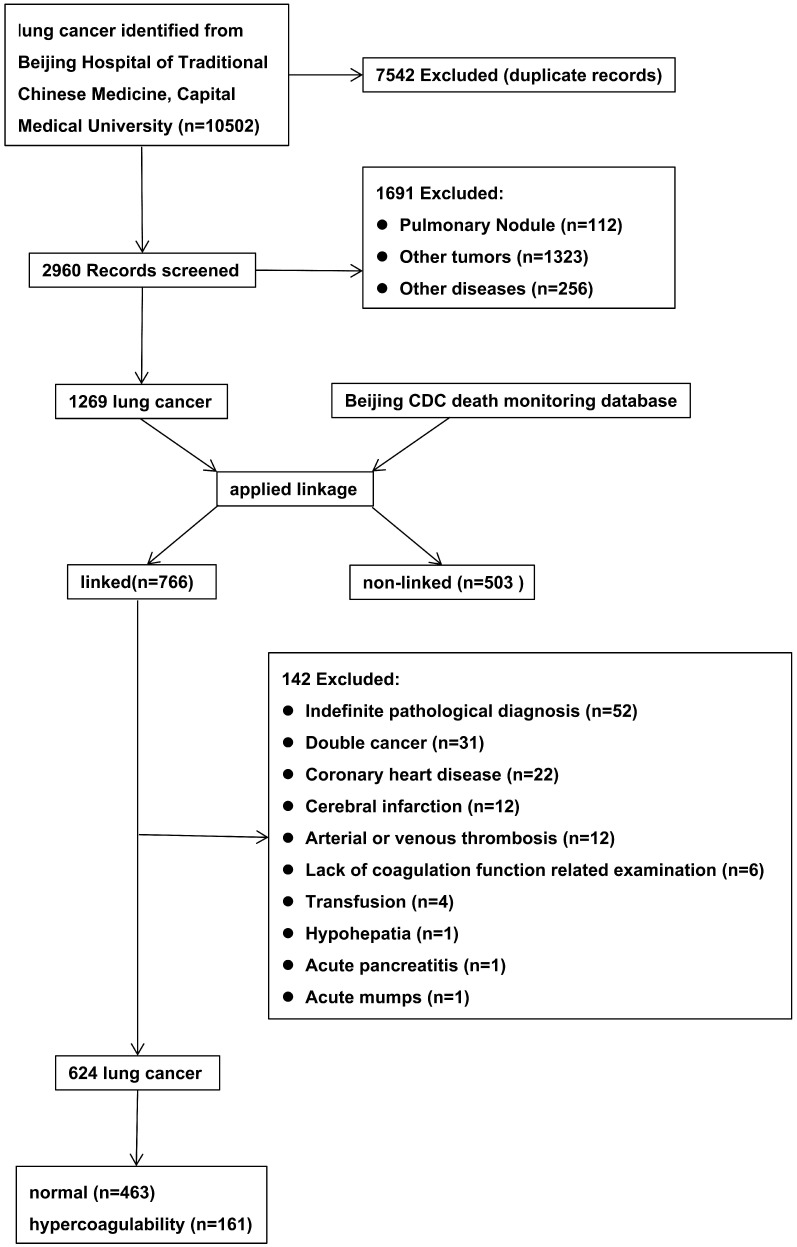
Flow diagram for selection of lung cancer.

The initial search retrieved 10,502 records. After meticulous inspection of the patient information, 624 inpatients from 2010-2017 were enrolled in this study. The patient selection process was shown in Figure 1 and the characteristics of the included patients were shown in Table 1. There were no significant differences in gender, process, smoking status, alcohol consumption, tumor location, or pathological type between LC patients with hypercoagulability (hypercoagulability group) and those without (normal group) (*P* > 0.05 for each). There were statistically significant differences in age, body mass index (BMI), clinical stage, carcinoembryonic antigen level (CEA), neuron-specific enolase level (NSE), cytokeratin 19 fragment level (Cyfra211), platelet-to-lymphocyte ratio (PLR), and neutrophil-to-lymphocyte ratio (NLR) between the groups (*P* < 0.05 for each).

### Kaplan-Meier analysis for overall survival

**Figure 2:**
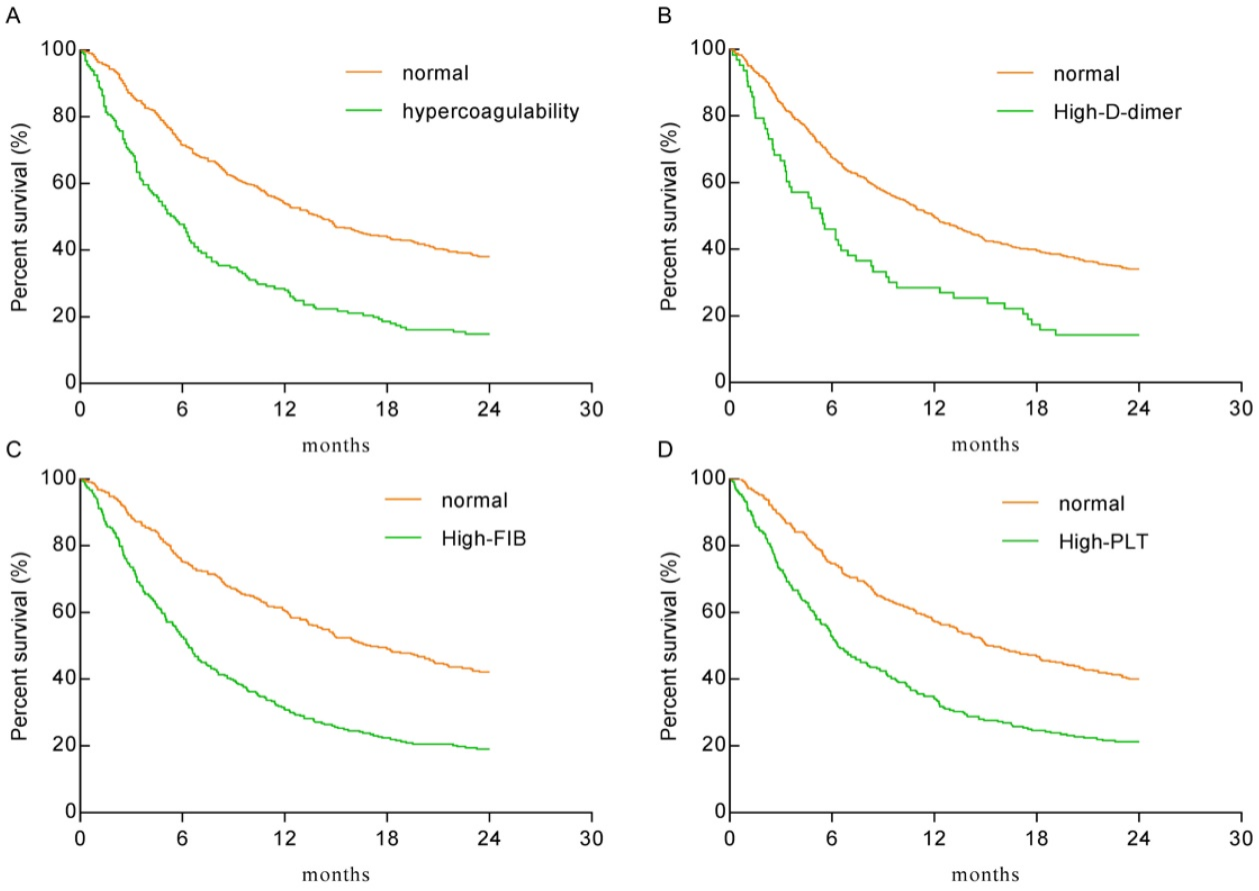
** Kaplan-Meier plots for the total population. A.** The hypercoagulability group VS the normal group (HR: 2.128, 95% CI: 1.664-2.721; *P* < 0.0001). **B.** The high-D-dimer group VS the normal group (HR: 1.922, 95% CI: 1.325-2.786; *P* < 0.0001). **C.** The high-FIB group VS the normal group (HR: 2.074, 95% CI: 1.697-2.534; *P* < 0.0001). **D.** The high-PLT group VS the normal group (HR: 1.851, 95% CI: 1.515-2.263; *P* < 0.0001).

As shown in Figure 2, the hypercoagulability group (HR: 2.128, 95% CI: 1.664-2.721), the high-D-dimer group (HR: 1.922, 95% CI: 1.325-2.786), the high-FIB group (HR: 2.074, 95% CI: 1.697-2.534), and the high-PLT group (HR: 1.851, 95% CI: 1.515-2.263) had significantly lower overall survival (OS) than the normal group(*P* < 0.0001 for each). The median survival times of the hypercoagulability and normal groups were 5.5 months and 14.1 months, respectively.

### ROC curves of PLT, D-dimer, FIB and hypercoagulability

**Figure 3:**
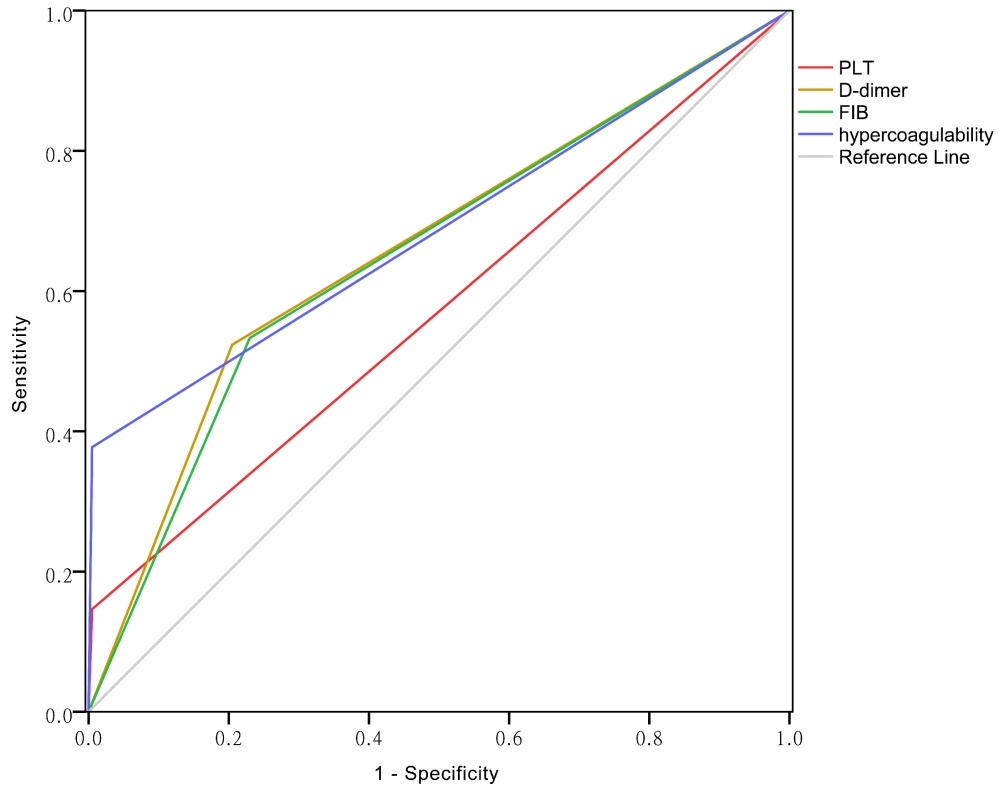
The ROC curves of PLT, D-dimer, FIB and the hypercoagulability.

The area under the curve (AUC) reflects predictive accuracy 14. As shown in Figure 3, the AUCs of PLT, D-dimer, FIB and hypercoagulability were 0.571 (95% CI: 0.525-0.617), 0.659 (95% CI: 0.615-0.704), 0.652(95% CI: 0.607-0.696), and 0.686 (95% CI: 0.646-0.727), respectively (*P* < 0.01 for each).

### Univariate analysis for overall survival

**Figure 4:**
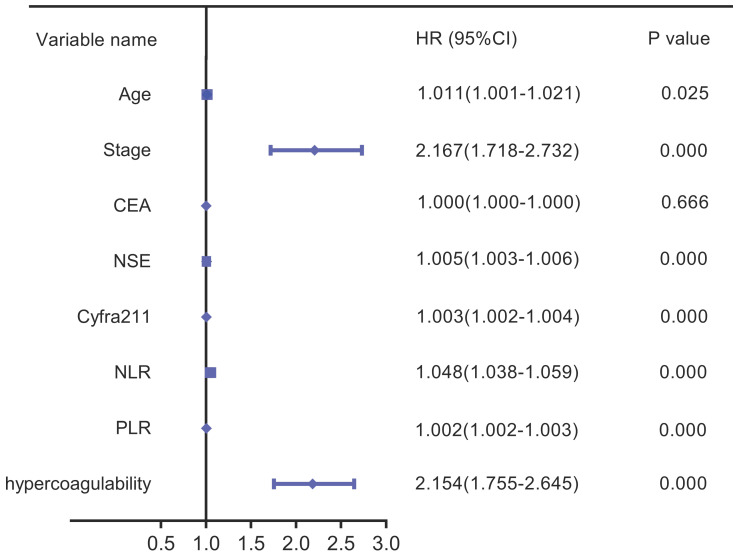
Univariate forest plot for overall survival.

Based on the analysis of the patient characteristics, we further analyzed the relationships between prognosis and age, stage, CEA, NSE, Cyfra211, NLR, PLR, and hypercoagulability. Univariate analysis revealed that hypercoagulability was significantly associated with decreased OS (HR: 2.154, 95% CI: 1.755-2.645, *P*=0.000). Additionally, age, stage, NSE, Cyfra211, NLR, and PLR were significantly associated with poor OS in LC (Figure 4).

### Multivariate analysis of overall survival

As summarized in Table 2, a multivariate Cox regression analysis was performed using hypercoagulability, age, stage, PLR, NLR, CEA, NSE, and Cyfra211 to investigate the independent prognostic factors for OS of LC. Hypercoagulability was still an independent prognostic factor associated with poor OS (HR: 1.591, 95%CI: 1.262-2.005, *P*=0.000), along with stage, PLR and NSE.

### Factors associated with hypercoagulability in LC patients

As shown in Table 1, there were statistically significant differences in age, BMI, clinical stage, CEA, NSE, Cyfra211, PLR, and NLR between the groups (*P* < 0.05 for each). We conducted logistic regression analysis to examine the correlation between these variables and hypercoagulability. The results showed that clinical stage (odds ratio [OR] = 3.672, *P* < 0.05), Cyfra211 (OR = 1.007, *P* < 0.05), and the PLR (OR = 1.006, *P* < 0.05) were positively correlated with hypercoagulability (Table 3).

### Clinical stage, Cyfra211, and the PLR predict hypercoagulability

**Figure 5:**
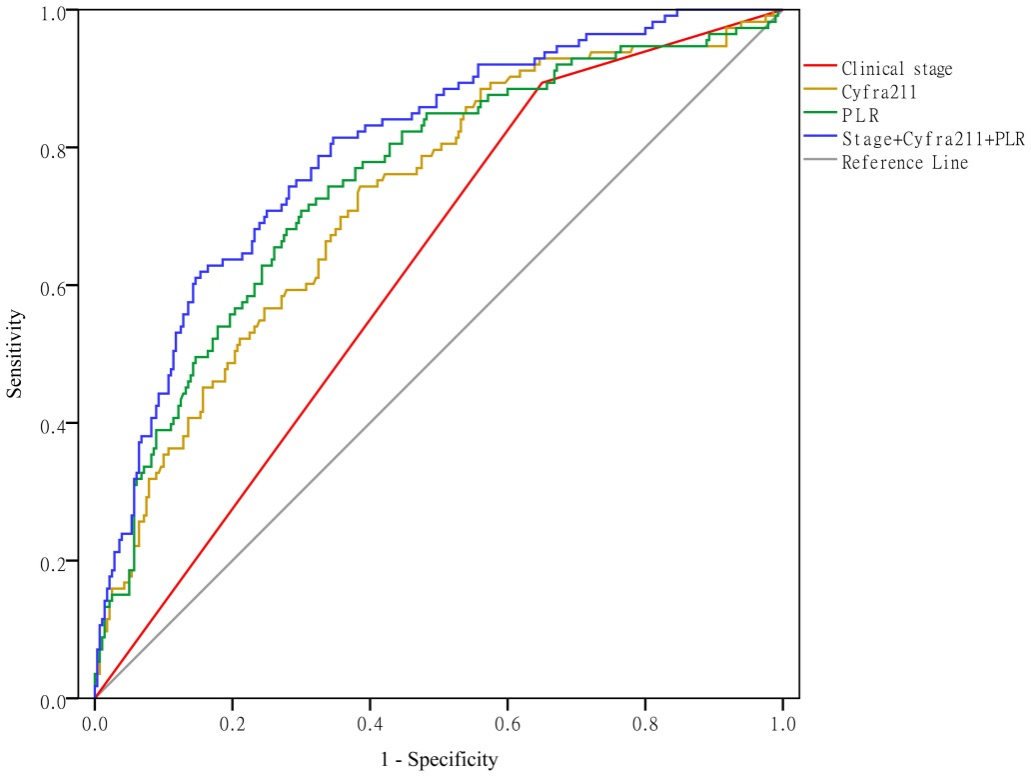
Clinical stage, Cyfra211, and the PLR predict hypercoagulability.

As shown in Figure 5, when using clinical stage, Cyfra211, and the PLR alone to predict hypercoagulability, the AUCs were 0.622 (95% CI: 0.564-0.679, *P* < 0.01), 0.724 (95% CI: 0.669-0.779, *P* < 0.01), and 0.750 (95% CI: 0.695-0.804, *P* < 0.01), respectively. When using these variables together to predict hypercoagulability, the AUC was 0.797 (95% CI: 0.749-0.845, *P* < 0.01).

## Discussion

Metastasis is the leading cause of LC-related death. The pathological mechanism of LC metastasis is extremely complex. Evidence suggests that the coagulation-fibrinolysis system is closely related to the progression of LC and that activation of coagulation is common in LC 15,16. A previous prospective study demonstrated that LC patients had higher blood coagulation parameters than healthy volunteers, including PLTs, FIB, thrombomodulin, and D-dimer 17. A retrospective study in Poland found that the frequency of thrombocytosis in surgically treated patients with non-small cell lung cancer (NSCLC) was 10.2% 18. Another study of small cell lung cancer found that 64.6% of patients had high levels of D-dimer 19. A retrospective study of 856 patients with NSCLC found that 43.6% of patients had elevated FIB 20. In this study, 161 patients (25.8%) had hypercoagulability. Although the mechanism of hypercoagulability in LC has not been elucidated, some associated factors have been reported. Tissue factor is the cell membrane receptor of the serine proteinase coagulation factor VII, whose physiological function is to trigger the activation of the coagulation cascade and increase blood coagulation 21. It is the most widely studied tumor coagulation factor and is considered to play a central role in the pathophysiology of hypercoagulability 22. In addition, inflammatory factors play a significant role in coagulation. These factors not only damage endothelial cells and promote angiogenesis, but also increase the expression of cell surface adhesion molecules 23. Activation of the contact system 24 and thrombocytosis 25 are closely associated with the development of hypercoagulability in cancer.

We demonstrated that hypercoagulability was an independent factor affecting the prognosis of LC and the predictive power of hypercoagulability was better than that of any single coagulation indicator. The hypercoagulability group had significantly lower OS than the normal group. Studies have previously shown that hypercoagulability is a common characteristic of cancer patients and is closely correlated with tumor growth and metastasis 26,27. In addition, hypercoagulability predisposes patients to venous thromboembolisms, pulmonary embolism, and disseminated intravascular coagulation, which affect the prognosis of LC patients. The result of the logistic regression analysis showed that clinical stage, Cyfra211 and the PLR were positively correlated with hypercoagulability in LC patients. The relationship between clinical stage and thrombocytosis has been observed in previous studies 28,29. Numerous studies have reported the prognostic significance of the PLR in cancer 30,31.

The aim of this retrospective study was to explore the clinical regularity and prognosis of LC patients with hypercoagulability. The main strength of this study is that it is a real-world study with a relatively large sample size and objective information. Furthermore, this is the first study to apply a hypercoagulation model to analyze the relationship of hypercoagulability and prognosis in patients with LC. This study provides a new perspective for better understanding of the relationship between hypercoagulability and prognosis in LC. Nevertheless, this study has several limitations. The study used a retrospective design, which might induce potential confounding factors. The analysis was limited due to the lack of data on important aspects of treatment, such as surgery, chemotherapy, radiotherapy, targeted therapy, and immunotherapy. Therefore, it is necessary to validate this conclusion in prospective multicenter studies that include stratified analyses according to patient characteristics.

## Conclusion

Hypercoagulability is an independent factor associated with poor OS and could be a prognostic factor for LC.

## Figures and Tables

**Table 1:** Main characteristics of all the patients included in the study

Characteristics of cases	Hypercoagulability	Normal	χ^2^/Z	*P*
**Age (years)**			6.825	0.033
18-45	8 (5 %)	9 (1.91%)		
46-65	60 (37.3%)	213 (46.0%)		
66-85	93 (57.8%)	241 (52.1%)		
**Process (years)**			4.736	0.192
<1	116 (72.0%)	297 (64.1%)		
≥1-<2	19 (11.8%)	69 (14.9%)		
≥2-<5	21 (13.0%)	66 (14.3%)		
≥5	5 (3.1%)	31 (6.7%)		
**Gender**				
Man	95 (59.0%)	276 (59.6%)	0.018	0.893
Woman	66 (41.0%)	187 (40.4%)		
**Smoke**			0.128	0.721
Yes	84 (52.2%)	234 (50.5%)		
No	77 (47.8%)	229 (49.5%)		
**Drink**			0.46	0.831
Yes	50 (31.1%)	148 (32.0%)		
No	111 (68.9%)	315 (68.0%)		
**Tumor location**			0.461	0.497
Left lung	71 (44.1%)	190 (41.0%)		
Right lung	90 (55.9%)	273 (59.0%)		
BMI*			6.981	0.031
<18.5	9 (7.5%)	20 (6.8%)		
≥18.5-≤23.9	80 (66.7%)	157 (53.8%)		
≥24	31 (25.8%)	115 (39.4%)		
**Pathological type**			3.766	0.288
adenocarcinoma	109 (67.7%)	291 (62.9%)		
squamous carcinoma	33 (20.5%)	96 (20.7%)		
small cell lung cancer	14 (8.7%)	66 (14.3%)		
other	5 (3.1%)	10 (2.2%)		
**Clinical stage**			35.479	0.000
I-III/limited stage	18 (11.2%)	167 (36.1%)		
IV/extensive stage	143 (88.8%)	296 (63.9%)		
CEA (ng/ml)	114.37±455.61	59.24±159.06	-3.288	0.001
NSE (ng/ml)	34.22±53.28	24.97±38.46	-2.757	0.000
Cyfra211 (ng/ml)	37.46±85.01	12.71±39.24	-7.203	0.000
PLR	315.00±205.06	196.38±131.91	-8.848	0.000
NLR	6.49±4.79	4.46±5.15	-7.022	0.000

*For some reasons, some patients' height or weight data are missing in the HIS system. Therefore, the BMI analysis in this article is only applied to 412 patients.

**Table 2:** Multivariate Cox regression analysis for overall survival

Covariates	B	S.E.	Wald	Sig.	Exp(B)	95% CI for EXP(B)	
						Lower	Upper
Age	0.010	0.005	3.755	0.053	1.010	1.000	1.020
Stage	0.545	0.126	18.660	0.000	1.725	1.347	2.209
PLR	0.001	0.000	6.515	0.011	1.001	1.000	1.002
NLR	0.019	0.010	3.401	0.065	1.019	0.999	1.040
CEA	0.000	0.000	1.185	0.276	1.000	1.000	1.000
NSE	0.004	0.001	18.468	0.000	1.004	1.002	1.005
Cyrea211	0.001	0.001	3.641	0.056	1.001	1.000	1.003
Hypercoagulability	0.464	0.118	15.421	0.000	1.591	1.262	2.005

**Table 3:** Logistic regression analysis of hypercoagulability and Age, BMI, Clinical stage, CEA, NSE, Cyfra211, PLR, NLR

Dependent	Covariates	B	S.E.	Wald	Sig.	Exp(B)	95% CI for EXP(B)	
							Lower	Upper
Hypercoagulability	Clinical stage	1.301	.364	12.761	.000	3.672	1.799	7.497
	Cyfra211	0.007	.003	6.311	.012	1.007	1.002	1.012
	PLR	0.006	.001	32.995	.000	1.006	1.004	1.007
	Constant	-4.752	.722	43.274	.000	.009		

## Abbreviations

LC: lung carcinoma
PLT: platelet
PFS: progression-free survival
BHTCM: Beijing Hospital of Traditional Chinese Medicine
HIS: Hospital Information System
FIB: fibrinogen
ROC: receiver operative characteristic
BMI: body mass index
HR: hazard ratio
CI: confidence interval
OS: overall survival
CEA: carcinoembryonic antigen
NSE: neuron-specific enolase
Cyfra211: cytokeratin 19 fragment
OR: odds ratio
PLR: platelet-to-lymphocyte ratio
NLR: neutrophil-to-lymphocyte ratio
AUC: area under the curve
NSCLC: non-small cell lung cancer
